# Augmented Ensemble Model (AEM) for health trends prediction on social networks

**DOI:** 10.1371/journal.pone.0323449

**Published:** 2025-06-05

**Authors:** Sonia Saini, Ruchi Agarwal, S.P. Singh, Punit Gupta, Ankit Vidhyarthi, Rohit Verma

**Affiliations:** 1 Associate Consultant, Tata Consultancy Services, Noida, India; 2 Professor, Computer Applications Department, JIMS Engineering Management Technical Campus, Greater Noida, India; 3 Department of Computer Science and Engineering, BIT Mesra, Ranchi, India; 4 Department of Computer Science and Engineering, Pandit Deendayal Energy University, Gandhinagar, India; 5 Department of Computer Science and Engineering, Jaypee Institute of Information Technology, Noida, India; 6 Technological University Dublin, Dublin, Ireland; Sreenidhi Institute of Science and Technology, INDIA

## Abstract

Social Media has given an exponential rise to an ever-connected world. Health data that was earlier viewed as hospital records or clinical records is now being shared as text over social media. Information and updates regarding the outbreak of a pandemic, clinical visit results, general health updates, etc., are being analyzed. The data is now shared more frequently in various formats such as images, text, documents, and videos. With fast streaming systems and no constraints on storage spaces, all this shared rich media data is quite voluminous and informative. For shared health data such as discussions on ailments, hospital visits, general health well-being updates, and drug research updates via official Twitter handles of various pharmaceutical companies and healthcare organizations, a unique level of challenge is posed for analysis of this data. The text indicating the ailment often varies from proper medical jargon to common names for the same, whereas the intent is the same in predicting the disease or ailment term. This paper focuses on how we can extract and analyze health-related data exchanged on social media and introduce an Augmented Ensemble Model (AEM), which identifies the frequently shared topics and discussions about health on social networks, to predict the emerging health trends. The analytical model works with chronological datasets to deduce text classification of topics related to health. This Hybrid Model uses text data augmentation to address class imbalance for health terms and further employs a clustering technique for location-based aggregation. An algorithm for health terms Word Vector Embedding model is formulated. This Word Vector model is further used in Text Data Augmentation to reduce the class imbalance. We evaluate the accuracy of the classifiers by constructing a Machine Learning pipeline. For our Augmented Ensemble Model, the Text classification accuracy is evaluated after the augmentation using a voting ensemble technique, and a greater accuracy has been observed. Emerging health trends are analyzed via temporal classification and location-wise aggregation of the health terms. This model demonstrates that a Text Augmented Ensemble Machine Learning approach for health topics is more efficient than the conventional Machine Learning classification technique(s).

## 1. Introduction

Social Networks have given a new and exciting study dimension to analytics on health-related terms and topics. Social media is increasingly providing tools to share information with health care professionals and people discussing health on social networks. Health care professionals discuss various health care policies and health care practice issues, promote a healthy lifestyle, engage with the public, educate and interact with the patients, the caregivers, and the relevant people of society [1]. While existing research used offline datasets or Medical Records. Nowadays, with the extensive use of social media to discuss and share health-related information, it has become possible to gather meaningful data for analytics on health or healthcare topics. However, when considered from a time-based perspective like year-wise or month-wise, the varying data presents challenges to predictive analysis. To evaluate the scenario of determining which health topics or terms are discussed the most across different time sets, we must devise a more refined approach than just be using conventional machine learning techniques. We need to espouse a model that “normalizes” the classes’ health terms for better health term analysis. Health term classes under predictive analysis are rendered as a well-defined set of known health entities. Extracting various health topics from social networks presents a unique challenge in narrowing key health terms. The textual representation of the same health term, disease, or ailment may vary vastly across the corpus under study. This data can give rise to n different classification classes for the health terms with the same connotation. The commonly available word vector embedding is of little or no use for medical terms. Hence, a need arises for a data augmentation technique to be devised and employed, which can augment health terms text corpus so that a subsequent text classification yields actual True-Positive results.

Data Augmentation is a strategy to generate more data from the existing dataset via operations such as transformation, shuffling, replacement, substitution, etc. Text data augmentation applies to Natural Language Processing problems and is done to augment classes to reduce overfitting. There are various types of text augmentation techniques such as similar word method, synonym method, interpolation method, extrapolation method, and addition of random noise [2].In the case of Text Data Augmentation, there can be several transformations like “Thesaurus”, “Word Embedding”, “Back Translation”, “Contextualized Word Embedding based on K-Nearest Neighbors and Cosine similarity”, “Text Generation”, and “Text Substitution” [3,4].

For text analysis, researchers have widely used Natural Language Processing and information retrieval techniques such as text extraction, syntax analysis, methods used for getting descriptions, text via metadata or annotation processing, etc. We can further analyze the text with Machine Learning techniques to arrive at meaningful insights.

The work aim to propose an improved model using data augmentation and ensemble techniques to improve the accuracy of existing machine learning model. This allows to identify more complex pattern in the data using word2vec and Text data augmentation techniques. The objective of this work is to train the model graph algorithms model using node embedding techniques to improve the performance of existing model. Where graph algorithm techniques allows to identify and train better pattern in data which results better performance and precision.

The work is divided into 5 sections where section 2 discusses existing work from the field of Text data augmentation and Ensemble machine learning. Section 3 discusses the proposed Augmentation-based Ensemble Model for healthcare data with various steps performed. Section 4 showcases the experimental setup and result analysis of the proposed work as compared with existing works. Lastly section 5 discusses the conclusion and future scope of this work.

## 2. Related research

Data augmentation techniques are widely used in Convolutional Neural Networks in auguring the training of visual recognition systems by artificially increasing the number of training samples. Popular frameworks such as the Keras Deep Learning framework provide the ready built-in class for image data augmentation. Typically, data augmentation is done to provide for class deficits so that the model has well-balanced training data to learn well.

Often the social media datasets obtained for analysis will have a class imbalance. Prior research on text data augmentation (to address the class imbalance) has met with modest success. An augmentation of text data done at paragraph level achieved an improved classification accuracy of 10 percent. The paragraph order switch is not supposed to alter the semantic meaning of the text [5]. The text data augmentation for the NLP technique of Easy Data Augmentation (EDA) espoused four powerful operations like Synonym Replacement (SR), Random Insertion (RI), Random Swap (RS), and Random Deletion (RD). It demonstrated particularly strong results for smaller datasets [6]. When running on different training set sizes for Convolutional Neural Networks (CNNs) and Recurrent Neural Networks (RNNs), EDA yielded an average accuracy of 88.6 percent vs CNNs, RNNs without EDA providing an accuracy of 87.8 percent for classification. Work has also been done on pre-trained word embedding for biomedical words and sentences [7,8].

Ensemble Machine Learning is an approach in machine learning to tweak the machine learning model to predict improved accuracy. It involves many fold iterations over datasets to achieve a more refined accuracy score. Ensemble Machine Learning improves the algorithm accuracy and the robustness of the model. Ensemble methods accomplish this by addressing the bias and variance errors trade-off with respect to any model’s complexity and the errors produced [9]. It is regularly used in predictive analytics to improve prediction accuracy by blending multiple predictors [10].

For Ensemble machine learning, it was demonstrated that data diversity could be accounted for using an ensemble, and a Support Vector Machine (SVM) ensemble showed better classification [11]. It has also been pointed out in the research that an Ensemble Machine Learning based Analytical Model for health topics data from Social Network is better than gauging the accuracy using traditional Machine Learning techniques [12].

In another research, Random Forest was evaluated for text classification accuracy. Weighted Tree Random Forest was found to have the best text classification accuracy when compared with Support Vector Machine (SVM), Naïve Bayes (NB), k-Nearest Neighbors (kNN), Brieman’s Random Forest and Tree Selection method [13].

Linear Support Vector Machine (LSVM) has been widely acknowledged as one of the best text classification algorithms. Ensemble methods are extensively used to solve multi-label classification tasks. Ensemble learning techniques have already demonstrated superior accuracy in supervised learning machine learning tasks [14–19]. Boosting and Bagging are popular ensemble learning techniques. Boosting based ensemble being done along with bootstrapping as a precursor step, which may be required based on the dataset. Bagging, or bootstrap aggregation, is a process with three stages wherein we bootstrap the data, do collection, and combine predictions from various models. The Stacking technique and the Voting Ensemble with Majority or Minority Voting are also good choices for predicting the class labels. Most of the time, data have an uneven distribution of classes and thus presents an obstacle for accurate classification. Research has also focused on using a self-trained classifier’s ensembles to address data imbalance [20].

When there is a class imbalance, performance metrics can be chosen on class equality, class positivity in class-labels based prediction, and probabilities. In the case of the class labels being equally important, it is recommended to use the G-Mean performance metric. When less than 80–90% of the examples belong to the majority class, it is recommended to use the metric of accuracy [6]. In [21] summarizes the various ensemble learning approaches. In another research, Ensemble machine learning is used to predict early onset of diabetes [22]. In [23] Based on the lifestyle indicators, Ensemble machine learning is used to predict diabetes. The research was also carried out to diagnose Osteoporosis [24] and Diabetic Retinopathy detection [25].

For Health domain text data analysis, research has primarily focused on the medical field words, such as proper medical terms like arrhythmia, carcinoma-in-situ, diabetes mellitus, etc. These terms are more frequently used in medical transcriptions, notes, and reports and have lesser weightage in a social sharing context as most people are unaware of these scientific and thus uncommon terms. There is a need to implement a “health terms word vector embedding model” (common health terms used while posting on social media, such as cancer, diabetes, etc.). The below approach outlines the development of the same.

## 3. Proposed model

The problem under study is the analysis of health-related textual data to infer emerging health topics trends. This proposed model is used to predict various health ailments or health term classes with the use of ensemble machine learning. Social network data is an amalgamation of rich media with demographic differences. Data sets from a social network such as Twitter, which have textual attributes, act as a conducive medium for machine learning techniques to use ensemble learning. For a classifier, the quantification of results may mean striking a balance between bias and variance, the source of variance being a limited sample size. A large sample size, by comparison, could still yield unreliable results, but it would significantly reduce the variance of predictions. We can achieve more accurate predictions by reducing bias and variance. An ensemble of classification models and an ensemble of clustering models can be combined to minimize bias and variance for diverse temporal data from social networks.

We propose an Augmentation-based Ensemble Model (AEM) to reduce bias and variance. This model is trained as a final model using a Health terms Word Vector binary. The model’s augmentation aspect relates to the augmentation of text data, the objective of which is to minimize overfitting. By resolving the class imbalance, data augmentation works by down-sampling the majority class and up-sampling the minority class. Tag replacement and text swap substitution are two typical implementations used for text augmentation.

The proposed work is divided into following phases:

- Data Collection
- Cleaning and pre processing
- Model designing and training

1. Data collection

The dataset is Twitter dataset of tweets about health/diseases and similar terms having Health Terms in tweets for health terms frequency-based trend detection. For information retrieval of the “country of origin” of the tweet, we employed an approach of co-ordinates based geocode lookup and further geocode to country mapping. The dataset is collected under free licence of twitter where twitter API allows you to collect data based on keywords. The dataset is free and open source to at the link given below:

https://github.com/punit-gupta/dataset_heart_t

Each row of the dataset has these attributes associated with it:

**diseaseTerm** is the disease Term class derived from the hashtag processing of tweet Text

**tweetText** is the “cleaned” tweet text

**dateOfTweet** is the date of creation of the tweet on Twitter, important from a chronological analysis perspective.

**countryOfTweet** is the country which has been derived by a **reverse_geocode** function, which outputs country name when provided with geo-coordinates as the input.

The above Table 1 describes the curated data from the Twitter Dataset. The **dateOfTweet** and **countryOfTweet** are derived after pre-processing the original tweet text having a date as timestamp and geo-coordinates out of which the respective country name is derived. The data includes 365338 rows for anxiety data and 472915 rows for depression related tweets which will include multiple diseases.

**Table 1:** The curated data from the Twitter Dataset.

diseaseTerm	tweetText	dateOfTweet	countryOfTweet
bleedingdisorders	These groups have joined forces in the bleedingdisorders community to advance hemophilia research	03-01-2014	United States
hemophilia	Personally Paced Zumba good exercise for hemophilia and bleedingdisorders Hemophilia	07-01-2014	United States
cancer	Minnesota Woman Beats Cancer with Measles Vaccine cancer bloodcancer	15-05-2014	Netherlands

1. Cleaning and pre processing

This phase includes preprocessing and cleaning twitter data with following operation:

- Remove Duplicates tweets
- Missing Values removal
- Removing URL, Hastags and Special Characters and Punctuation
- Remove stop words like “the, is, and, a, an…” using NLTK library
- Tokenization of the tweet into words

Data Preprocessing was done to remove the text corpus of noise such as acronyms, emoticons, stop words, and other unwanted data. The experiment was run using Python 3.7, and the scikit learn and NLTK python library for Machine Learning. This steps helps to improve the quality of the data for training and removing unrelated and incomplete data.

1. Model designing

**Fig 1:**
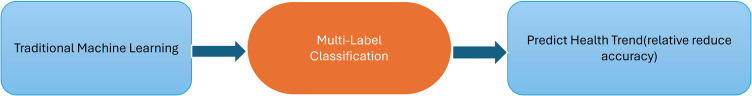
Conventional (Traditional) Machine Learning model.

In this phase the Augmented Ensemble Model is proposed with Text Data Augmentation for better classification as compared to tradition machine learning models as shown in Fig 1.

**Fig 2:**
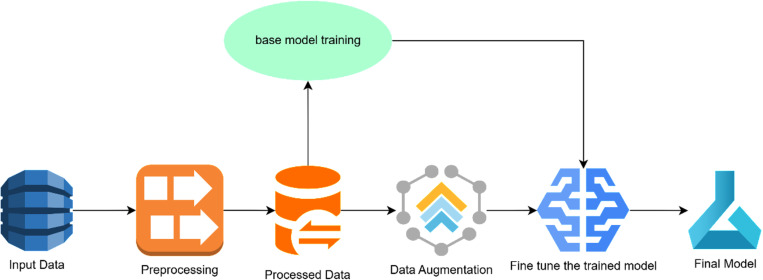
Proposed model architecture Augment Machine learning model.

Conventional (Traditional) Machine Learning model output has a relatively less accuracy for classification of the health terms text data. Fig 2 showcases the proposed model with AEM model for predicting health trends. Where in the proposed model after pre processing first a base model is trained then the existing model is tuned using Ensemble layer using augment data. The work is inspired by graph algorithm to find better pattern in existing data with complex relations ships.

**Fig 3:**
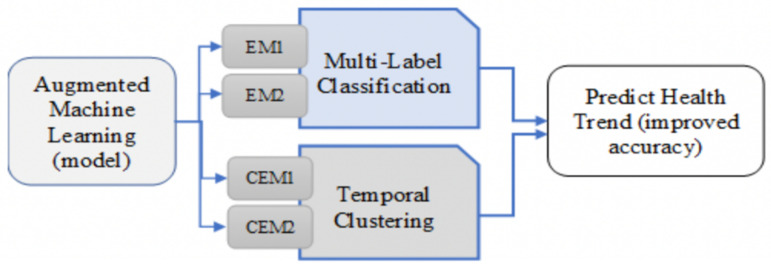
Augmented Ensemble Model (AEM).

The proposed model AEM model to improve the machine learning model which uses multi label classification and temporal clustering as shown in Fig 1.This model uses Augmented Ensemble Model (AEM) which can apply both an augmentation and an Ensemble approach to improve prediction accuracy. EM_1_ and EM_2_ are ensemble machine learning models. CEM1and CEM2 are ensemble models for clustering, which will cluster the topic wise multi-label classification by considering the tweets’ time as shown in Fig 3. The proposed AEM Model demonstrates refined accuracy in classification when we augment the text terms classes and then do an Ensemble-based classification. At Ensemble Layer “Voting Ensemble” is used for a probable improvisation in the classification accuracy.

### 3.1. Proposed approach to Text Data Augmentation

Text data augmentation aims to substitute disease terms text with equivalent predetermined and standardized disease term text to balance the overall disease classification. Class imbalance can render a machine learning model redundant in predicting accuracy. It may predict one class (disease term) with too high accuracy, whereas it may not predict other disease terms. It has been evident in standard classifier algorithms like Logistic Regression and Decision Trees, which cannot handle imbalanced classes. To boost the F1-Score for accuracy, we balance the disease terms by introducing Text Data Augmentation as a precursor part of this model before the corpus is further subject to ensemble learning-based accuracy enhancements. The following table explains the rationale for employing disease or Health Term Text Data Augmentation (HTTDA) in our model:

In Table 2, T_f_ (term word) is the transformation function applied to transform the term word. S_**f**_ (term word) is the substitution function applied to substitute the term word with an appropriate designated class word. As is evident from Table 1, the two disease terms text imply the same ailment but being textually different, these give rise to two separate classes for the model. After applying text data augmentation, the standardized disease terms text result in a controlled set of the classes for further text classification. We used a Python-based Text Data Augmenter for achieving this transformation.

**Table 2:** Health Term Text Data Augmentation (HTTDA) transformation for reduction to n-Classes.

Exhibit	Original Tweet text (Non- standardized)	Apply HTTDA transformation (∀ Med Terms)	Tweet disease term class (Non-standardized)	Resultant Tweet disease term class (standardized)
A	10 things every adult with #diabetes should know	10 things every adult with #diabetes should know	**diabetes**	**diabetes**
B	New drug trials show promise in #leukemia related ailments	New drug trials show promise in #leukemia related ailments (T_**f**_ (Leukemia) ◊ S_**f**_ (blood cancer) ◊ cancer)	**leukemia**	**cancer**

**Fig 4:**
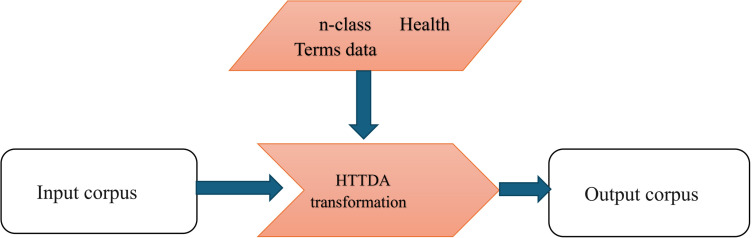
Health Terms Text Data Augmentation with the augmentation technique of “word substitution”.

The following diagram depicts the workflow for the HTTDA transformation:

Above Fig 4 depicts the Health Terms Text Data Augmentation workflow with the augmentation technique of “word substitution.” For the data augmentation technique of text substitution, we use the Word2Vec model with Gensim as the transformation effecting framework [26]. Using Gensim, the Word2Vec algorithms include skip-gram, and Continuous-Bag-of-Words (CBOW) frames for word embedding. Gensim allows for a selection between these two, along with various attributes like dimensionality of vectors and stipulations for a minimum count-based inclusion. Our approach to text data augmentation uses the **Natural Language Processing (NLP) Aug** Textual Augmenter python package with the option of **word augmentation with text substitution by the HTTDA word2vec similarity.**

The algorithm for the proposed unique “*Health Terms Word Vector Embedding Model*” using Gensim (HTWV Model) is stated as below:

**Algorithm 1**. Creating a pre-trained word embedding for Health Terms data for training and using a word embedding on Health Terms in the social media text.

1. Predetermined text of Health Terms (common terms used in social media)
2. Pre-processing of the text of Health Terms (cleaning, stop-word removal, symbols, links removal)
3. Generate Word2Vec embedding model from a text corpus of health terms
4. Train Word embedding Health Term Word Vector(HTWV) Model
5. Do intrinsic evaluation if required.
6. Apply the trained model to target data
7. Use NLP Aug with the Word2Vec model for text substitution for various classes determined in target data.
8. Get result text corpus with only n  deterministic health term classes.

Intrinsic evaluation is the introduction of a synthetic metric. For the intrinsic evaluation in the above algorithm, a popular word aggregation, called WordNet, has been used as a reference for semantic analysis of the word embedding. The most frequent nouns and verbs are extracted and compared to the clusters generated from the word embeddings.

# model_type: **Word2Vec**

aug = naw.WordEmbsAug(

model_type = **‘word2vec’**, model_path=model_dir+**’HealthTerms-vectors.bin**’, action = “**substitute**”)

augmented_text = aug.augment(text)

print(“Original:”)

print(text)

print(“Augmented Text:”)

print(augmented_text)

*Case 1*

***Original:***

*New drug trials show promise in* ***bloodsugar*** *related ailments*

***Augmented Text:***

*New drug trials show promise in* ***diabetes*** *related ailments*

*Case 2*

***Original:***

*New medical method for treating* ***skin cancer*** *shows improved results*

***Augmented Text:***

*New drug trials show promise in* ***cancer*** *shows improved results*

**Fig 5:**
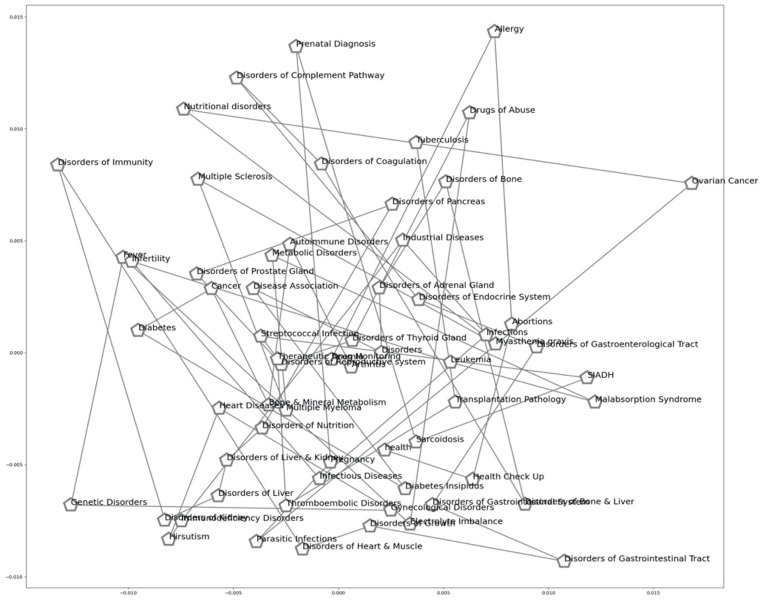
The PCA Health Terms Scatter plot of the devised model.

In order to visualize how the health terms linked semantically appear vis-à-vis the Key Class for the Health Terms, we plot the Principal Component Analysis graph to see the cosine similarity of the health terms. The PCA Health Terms Scatter Plot of the devised model is illustrated by the Fig 5 below.

The PCA Health Terms Scatter Plot of the devised model is illustrated by the Fig 5:

## 4. Experiment and results

The experiment uses text augmented data and does a multi-label classification. We predict text data in terms of various health classes. Dataset was taken from Twitter consisting of approximately 2 Lakh (0.2 million) tweets related to health terms and about 60 percent of which necessarily had the location. The disease term (class for the classification) has been derived from the *Tweet’s Hashtag (#) processing*. The tweet’s time, required from a chronological perspective for temporal clustering, is readily derived from when the tweet was created on Twitter. The experiment is performed using twitter data as discussed in section 3 where the total size of data after cleaning is 828002 rows. Where the training ad testing set is divided into 70–30 ration with random state as 42. The test were performed on Colab High computing environment with 12 GB RAM and 71 Gb memory and

### 4.1. Results

**Fig 6:**
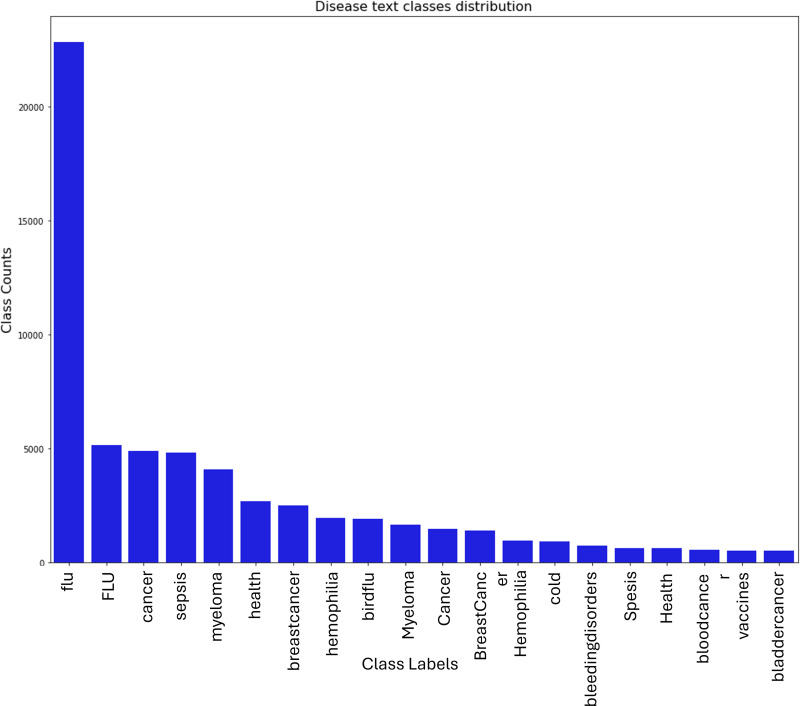
The Disease Text class distribution before applying the HTTDA word vector.

**Fig 7:**
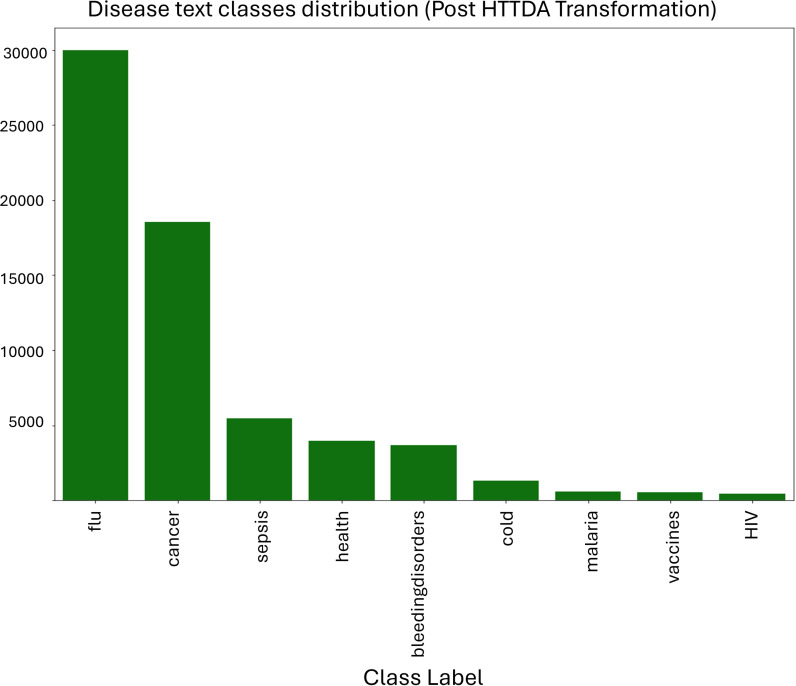
The disease text class distribution after the application of text data augmentation.

The figures below show that while for many health terms, the corpus indicates one category per tweet, the count of two, three, and four categories per tweet was also observed. The distribution of health terms before applying text data augmentation is shown in Fig 4 below:

In Fig 6, which has been obtained before Data Augmentation using the Health Terms Text Data Augmentation (HTTDA) Word Vector model, we observe the abnormality of the class counts for “health terms” for specific ailments. The above classification does not account for ***analogous disease text classes***. For example, “flu”, “Flu”, “bird flu” should all map to a group class “flu”. Similarly, “cancer”, “myeloma”, “hemophilia”, “Cancer”, “blood cancer”, “bladder cancer”, “breast cancer” should all map to a group class “cancer”. After applying the HTTDA transformations on disease term text classes, the resultant class label and class counts graph is as Fig 5:

Fig 7 shows the disease text classification after aggregating all the relevant health term classes under the parent Group Class. The text data from social media is quite expressive and unconstrained (not required to conform to any model). It can give rise to *n* different health terms with the same connotation. Therefore, it provides *n* different classes. It eventually results in too many classes for a large corpus where we have all types of health ailments/ disease terms under study.

### 4.2. Accuracy of the classifiers using machine learning pipeline

Post the Text Data Augmentation; we evaluated the accuracy of the text classification of various classifiers by constructing a machine learning pipeline. The results are as below (in Table 3)

**Table 3:** Accuracy of the classifiers using machine learning pipeline.

Machine Learning technique	Accuracy achieved
Logistic Regression	0.44687378987816867
Random Forest	0.5150430269607346
XGBoost Classifier	0.4847487718426368
Results post convert to TF-IDF vectors using the Scikit Learn (skLearn) TfIdfVectorizer	
Pipeline with Tf-Idf & Logistic Regression - Accuracy	0.8770815644400223
Pipeline with Tf-Idf & RandomForest - Accuracy	0.8865393438362148
Pipeline with Tf-Idf & XGBoost - Accuracy	0.72964424623301

The Term Frequency-Inverse Document Frequency (TF-IDF) Vectorizer employs log values. It is different from the Count Vectorizer in that it computes the frequency of word per document weighted by the frequency of word per the whole corpus. The TF-IDF Vectorizer in sklearn gives us a matrix of Tf-idf of each word in each tweet, showing higher values for words specific to that tweet and low values (0) for words appearing throughout the tweet corpus. We observe that the TF-IDF Vectorizer with Random Forest classifier produces a comparatively good accuracy of 0.8865 (mentioned up to 4 decimal places). Higher accuracy can be attributed to Random Forest working well for datasets having high dimensionality.

### 4.3. Accuracy of classifiers using Ensemble Machine Learning

For Ensemble Machine learning, we use a text classification ensemble with a voting ensemble to determine if an ensemble performs better than a simple classifier. Voting Ensemble has Hard Voting (the class with the highest number of votes) and Soft Voting (summation and averaging of the probability vector for each predicted class for all classifiers).

We look at the macro F1 score, the weighted F1 score, and the micro F1 score. We deduce these metrics for a baseline classifier that uses TF-IDF Vectorizer and Logistic Regression with default parameters. We also do a stemming operation on the corpus and then use XGBoost with Stemming. Finally, we club the three classifiers’ results, Logistic Regression with stemming, Logistic Regression, and XGB with Stemming, into a Majority Voting Ensemble to deduce if the Majority Voting Ensemble performs better than these individual classifiers.

The formula gives the F1 score:


$$f1=2*\frac{\left(p*r\right)}{\left(p+r\right)}$$

The F1 score formula, where, p, and r, are precision and recall, respectively.

**Macro F1 score** Macro F1 score computes metrics (True Positive, False Negative, and False Positive) for each label individually and then finds their weighted mean. The Macro F1 score does not consider label imbalance.

**Micro F1 score** Micro F1 score calculates the metrics (True Positive, False Negative, and False Positive) globally by counting each metric’s total, respectively.

Table 4 below shows the Voting Ensemble’s performance against the Baseline, the Logistic Regression with Stemming, and the simple Logistic Regression. This study studies the performance of base model with AEM model, where majority voting ensemble moder performs the best. As shows in Table 4 the proposed model perform better than existing model because of the model is inspired by graph algorithm with node embedding techniques (word2vec) which allows the model to identify/classify complex relation ship among data and consider data as connected component where as existing machine learning are trained considering every row as independent entity. So this allows the proposed model to out perform the existing approaches.

**Table 4:** The F1 macro, weighted, and micro scores for various classifiers and a voting ensemble.

Classification approaches with score types	Accuracy score
Baseline	
macro:	0.32937636
weighted:	0.7626844
micro:	0.768946412
Logistic Regression with stemming	
macro:	0.406840115
weighted:	0.867596846
micro:	0.872431563
Logistic Regression	
macro:	0.076724006
weighted:	0.7840125
micro:	0.817714694
XGB with Stemming	
macro:	0.531318615
weighted:	0.879456055
micro:	0.882901909
Majority Voting ensemble	
macro:	0.604159343
weighted:	0.968377384
micro:	0.973652855

The above table shows F1 macro, weighted, and micro score for accessing the various classifiers’ performance and how the Majority Voting ensemble yields better classification accuracy.

**Fig 8:**
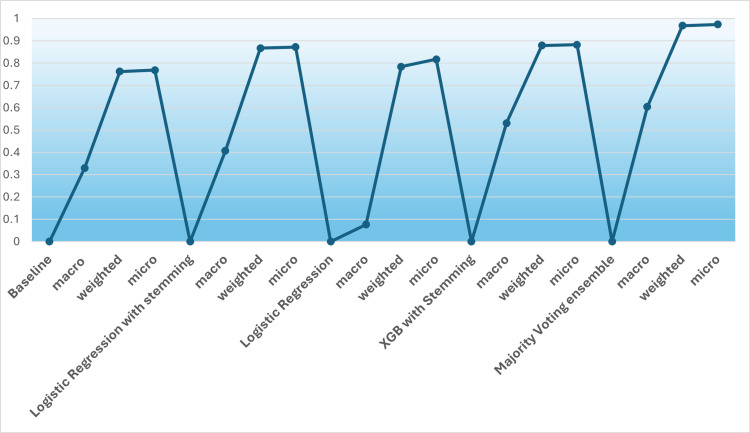
A comparative plot of the results of the performance of the classifiers for respective F1 scores (macro, weighted, and micro).

The performance of the classifiers can be visualized by displaying it as a chart below Fig 8.

The micro-F1 score gives us the accuracy of the classifier. As is evident from the above figure, the Voting Ensemble performs better for the health terms text classification accuracy than the Logistic Regression with Stemming or the XGBoost with Stemming, which themselves have a comparatively decent classification accuracy.

### 4.4. Temporal analysis

The below results show the tweets’ temporal distribution concerning the disease terms and count of tweets wherein multiple disease terms occur.

**Fig 9:**
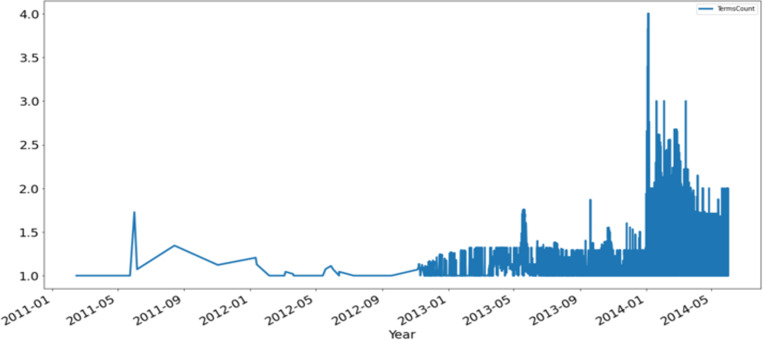
The plot of location-wise unique health terms per tweet over time.

Fig 9 describes the illustration of the count of more than one shows multiple disease terms from the tweet.

**Fig 10:**
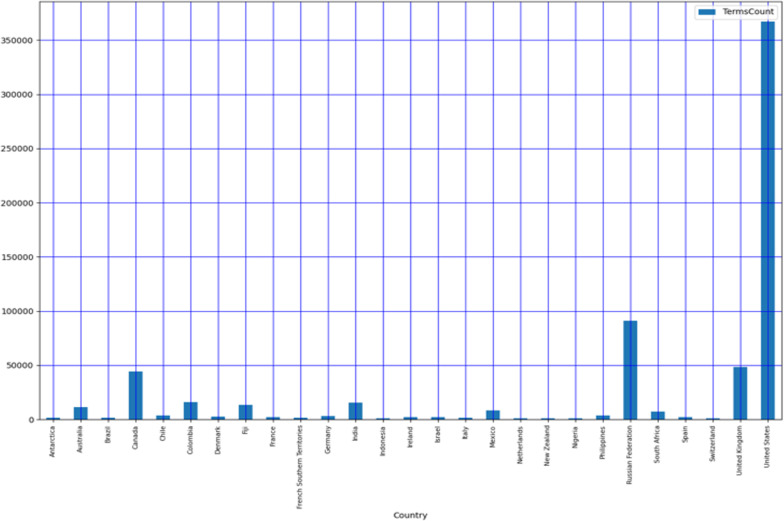
Country-wise aggregation of health terms from the tweet corpus.

**Fig 11:**
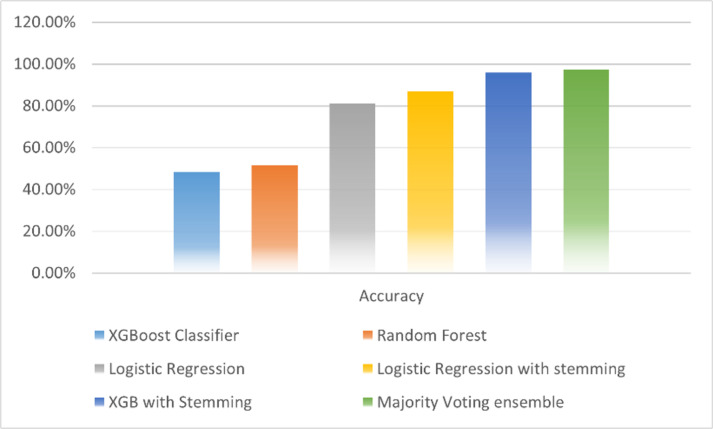
Comparative study of Accuracy as performance parameter.

It is evident from most of the disease health terms are from the U.S., followed by Russia, Canada, and the U.K., respectively as shown in Fig 10. The work has be compared with various existing machine learning methodologies to study an comparative analysis with proposed methodology. Table 5 shows the comparison of the proposed ensemble model with existing models where the comparison is done with logistic regression, XGBoost, random forest their variants. Where the proposed model out performs the exiting models with highest accuracy of 97.3%. Fig 11 shows the comparative study of accuracy of various models.

**Table 5:** Comparison with existing models.

Machine learning model	Accuracy
Logistic Regression with stemming	87%
Logistic Regression	81%
XGB with Stemming	96.8%
Majority Voting ensemble	97.3%
Random Forest	51.5%
XGBoost Classifier	48.4%

As the text data from social media is quite expressive and unconstrained (not required to conform to any model), it can give rise to n different health terms under the same connotation, thus giving n different classes, which ultimately result in a huge number of classes for a large corpus where we have all types of health ailments/ disease terms under study. In order to “normalize” this corpus with respect to the classes, we introduce a Group Class to class mapping with the mapping function as below:


$$\text{map}\left(\text{Gc}\to \text{C}\right)=\underset{i=1}{\overset{n}{\sum }}\;Ci+Gc$$

Map function for Group Class to Class mapping.

The above map function implies that i = 1 to n classes are mapped to Group Class and that every Group Class is also mapped to itself (as a class).

**Fig 12:**
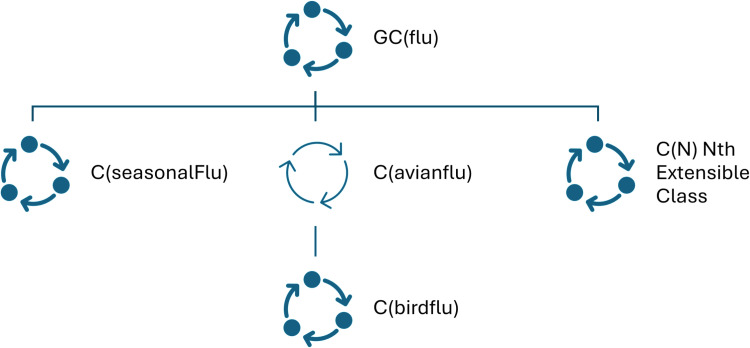
Illustrative Group Class to Class mapping for the Group Class “*flu*”.

**Fig 13:**
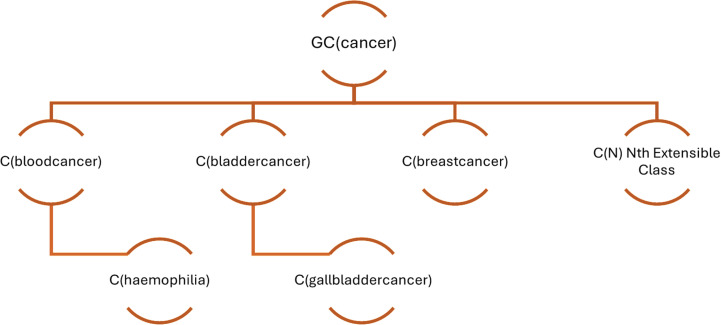
Illustrative Group Class to Class mapping for the Group Class “*cancer*”.

The Group Class (GC) to Class (C) hierarchy is demonstrated by the Figs 12 and 13.

The Group Class to Class map can be achieved by the mapping structure as below:

GC(flu): {{C(flu)}, {C(avianflu), C(birdflu)}}

GC(cancer): {{C(cancer)}, {C(bloodcancer), C(haemophilia)}, {C(bladdercancer), C(gallbladdercancer)}, {C(breastcancer}}

### 4.5. Research limitations

The limitation of this work is limited to dataset of 2 countries where the work can extended with study for more data. The proposed model is tested with limited models where the performance of the model can be tested with deep learning model and the present model can be tuned using various optimization algorithm to achieve best set of hyper parameters.

## 5. Conclusion and future scope

This paper proposed a model to infer emerging health trends using temporal data. This model works with social network text data and medical data obtained from hospitals or clinics, etc. We demonstrated an approach different from the traditional methods, such as label classification accuracy, which usually uses only a single classifier. We observed that we could increase classification accuracy by using a Text Substitution for data augmentation. We get better accuracy when we put health terms data through an ensemble of machine learning algorithms for classification to predict prominent health terms over a period. The text data augmentation model for Word Vectors, which is the basis for the data augmentation using the text substitution method, is open-ended in Group Classes. With this model, we have provided a baseline on which various types of data augmentation can further be applied for enhancing the accuracy of the classifier. In the further scope of this research, these Group Classes can be fine-tuned for a more refined data augmentation.
